# Fluoroquinolones and rifampin combination in the backdrop of heteroresistant tuberculosis

**DOI:** 10.1128/aac.01084-24

**Published:** 2025-01-16

**Authors:** Vanessa B. Vogensen, Sanjay Singh, Christopher J. Allende, David M. Engelthaler, Gunavanthi D. Boorgula, Tania A. Thomas, Marieke G. G. Sturkenboom, Onno W. Akkerman, Tawanda Gumbo, Shashikant Srivastava

**Affiliations:** 1 Department of Pulmonary Diseases and Tuberculosis, University Medical Center Groningen 10173 , Groningen, the Netherlands; 2 Division of Infectious Diseases, Department of Medicine, University of Texas at Tyler School of Medicine 675071 , Tyler, Texas, USA; 3 Translational Genomics Research Institute 525768 , Flagstaff, Arizona, USA; 4 Department of Medicine, University of Virginia 214841 , Charlottesville, Virginia, USA; 5 Department of Clinical Pharmacy and Pharmacology, University Medical Centrum Groningen, University of Groningen 666253 , Groningen, the Netherlands; 6 TB Center Beatrixoord, University Medical Center Groningen, Groningen, the Netherlands; 7 Quantitative Preclinical & Clinical Sciences Department, Praedicare Laboratories 581032 , Dallas, Texas, USA; 8 Department of Cellular and Molecular Biology, University of Texas Health Science Centre at Tyler 12341 , Tyler, Texas, USA; St. George's, University of London, London, United Kingdom

**Keywords:** fluoroquinolones, drug-resistance, *Mycobacterium tuberculosis*, hollow fiber model, pharmacokinetics/pharmacodynamics

## Abstract

The impact of heteroresistance on tuberculosis (TB) treatment outcomes is unclear, as is the role of different rifampin and isoniazid exposures on developing resistance mutations. Hollow fiber system model of TB (HFS-TB) units were inoculated with drug-susceptible *Mycobacterium tuberculosis* (*Mtb*) and treated with isoniazid and rifampin exposure identified in a clinical trial as leading to treatment failure and acquired drug resistance. Systems were sampled for drug concentration measurements, estimation of total and drug-resistant *Mtb*, and small molecule overlapping reads (SMOR) analysis for the detection of heteroresistance. In the second HFS-TB study, systems were inoculated with an isoniazid-resistant clinical strain and treated with various combinations of isoniazid, rifampin, moxifloxacin, and levofloxacin for 28 days. Linear regression and exponential decline models were used for data analysis. Suboptimal isoniazid and rifampin exposures failed to kill drug-susceptible *Mtb* in the HFS-TB. Standard susceptibility methods failed to detect drug resistance, but SMOR detected isoniazid and rifampin heteroresistance, as well as fluoroquinolone, to which bacilli were not exposed. *rpoB* mutations arising from low rifampin exposures were Q513K and H526N, whereas those from regimen adequate rifampin but low isoniazid concentrations were S531L. Moxifloxacin-rifampin combination sterilized the HFS-TB units inoculated with the isoniazid-resistant *Mtb* in 14 days compared with 21 days of treatment with levofloxacin-rifampin, with no further emergence of drug resistance. Early detection of isoniazid and rifampin heteroresistance could provide an opportunity to individualize the therapy and protect fluoroquinolones when added to the MDR-TB treatment regimen.

## INTRODUCTION

The emergence of antimicrobial resistance (AMR) during anti-tuberculosis therapy is a major global challenge (1–4). Drug-resistant tuberculosis (TB) is associated with high mortality and consumes a substantial portion of funds in high disease-burden settings (5). Heteroresistance is an important driver of acquired AMR in TB, defined as the minor resistant proportions of the overall *Mycobacterium tuberculosis* (*Mtb*) bacterial population in a patient (6). In the presence of heteroresistance treated with combination drug therapy, essentially, *Mtb* is treated with only one or two effective drugs, which leads to the drug-resistant subpopulation dominating the infection and eventually treatment failure (7). Overlapping read pairs sequencing approach has been used, as proof-of-principle to detect and quantify heteroresistance while reducing the sequencing error (8). Engelthaler and colleagues (9–11) further refined this by using the single-molecule overlapping read (SMOR) sequencing approach to develop it as a critical tool for detecting heteroresistance in *Mtb*.

The drug susceptibility testing of *Mtb* requires many weeks, and resistance to a drug is defined when the drug-resistant subpopulation crosses the threshold of >1% of the total bacterial population (12). Elsewhere using the preclinical hollow fiber model of tuberculosis (HFS-TB), we showed that both therapy failure and acquired AMR emerge due to inadequate drug exposure and pharmacokinetic (PK) variability, as seen in patients (13). In patients with drug-susceptible TB treated with standard combination therapy of isoniazid, rifampin, ethambutol, and pyrazinamide for 2 months, acquired AMR was developed in patients who had (i) rifampin peak (C_max_) of 0.81–4.62 mg/L and 0–24 h area under the concentration-time curve (AUC_0-24h_) of 7.24–10.83 mg*h/L, which, given only 30% penetration into cavities, translates to a C_max_ of 0.24–1.39 mg/L and AUC_0-24h_ of 2.17–3.25 mg*h/L in lung cavities, or (ii) isoniazid C_max_ of 0.88–8.6 mg/L and AUC_0-24h_ of 25.36–32.56 mg*h/L, which, given only 38% penetration into center of lung cavities, translates to a C_max_ of 0.33–3.27 mg/L and AUC_0-24h_ of 9.64–12.37 mg*h/L in cavities (14, 15). In a clinical study, direct measurements of drug concentrations inside MDR-TB lung cavities were isoniazid AUC_0-24h_ <4 mg*h/L, pyrazinamide AUC_0-24h_ <20 mg*h/L, and moxifloxacin AUC_0-24h_ <5 mg*h/L (14). In the present study, to test the hypothesis that heteroresistance due to subtherapeutic drug exposures can be detected early during the therapy using SMOR, HFS-TB units were treated with the drug exposures associated with treatment failure due to acquired AMR in patients.

Fluoroquinolone antibiotics (e.g., moxifloxacin and levofloxacin) are used to treat MDR-TB. The 6-month bedaquiline, pretomanid, linezolid, and moxifloxacin (BPaLM) regimen was introduced to treat rifampin-resistant TB and MDR-TB in patients not previously exposed to these antibiotics (16). However, reports of possible fluoroquinolone resistance in newly diagnosed patients with TB are emerging (17, 18). There are also concerns about possible drug-drug interaction, with rifampin leading to lower moxifloxacin AUCs (19). Here, we performed a second HFS-TB study to examine the efficacy of moxifloxacin versus levofloxacin in combination with rifampin in the presence of preexisting isoniazid resistance.

## RESULTS

### HFS-TB study with concentrations of rifampin and isoniazid associated with AMR

MICs of the laboratory strain *Mtb* H37Rv for isoniazid, rifampin, and pyrazinamide were recorded as 0.1 mg/L, 0.03 mg/L, and 25 mg/L, respectively. HFS-TB units were inoculated with *Mtb* H37Rv to explore the effect of subtherapeutic drug concentrations and AMR development. The isoniazid half-life (mean ± standard error) in HFS-TB was 5.43 ± 0.29 h, rifampin half-life was 5.06 ± 0.59 h, and pyrazinamide half-life was calculated as 11.06 ± 0.88 h. Regimen 1 was non-treated control. Regimen 2 achieved a pyrazinamide C_max_ of 66.08 ± 17.64 mg/L and AUC_0-24_ of 726.4 ± 165.7 mg*h/L, an isoniazid C_max_ of 6.36 ± 0.05 mg/L and AUC_0-24_ of 49.81 ± 3.08 mg*h/L, and rifampin C_max_ and AUC_0-24_ were below the limits of quantification of the liquid chromatography with tandem mass spectrometry (LC-MS/MS) method we used. Regimen 3 achieved a pyrazinamide C_max_ of 54.96 ± 14.87 mg/L and AUC_0-24_ of 634.7 ± 176.2 mg*h/L, an isoniazid C_max_ of 0.32 ± 0.05 mg/L and AUC_0-24_ of 2.02 ± 0.19 mg*h/L, and a rifampin C_max_ of 3.0 ± 0.06 mg/L and AUC_0-24_ of 20.89 ± 1.35 mg*h/L. Thus, HFS-TB was able to replicate drug concentrations associated with AMR in patients (7, 14).

**Fig 1:**
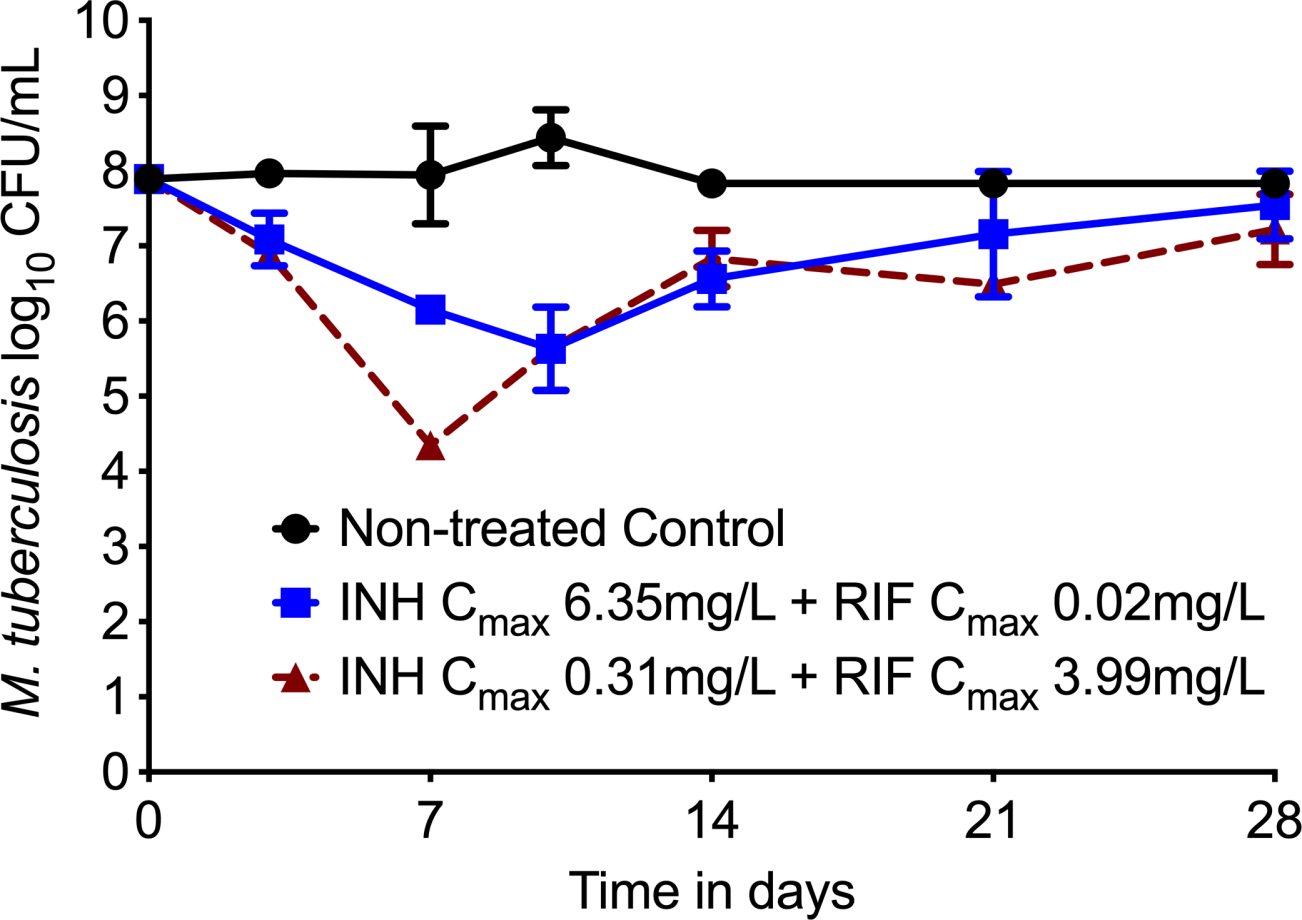
Comparison of the *M. tuberculosis* kill in HFS-TB treated with isoniazid and rifampin exposure associated with treatment failure in patients. Due to the high bacterial burden in the inoculum and the carrying capacity limitations, the *Mtb* burden remained static in the nontreated control systems (7.88 ± 0.08 log_10_ CFU/mL on day 0 versus 7.82 ± 0.03 log_10_ CFU/mL). In comparison, systems treated with isoniazid C_max_ 6.35 mg/L and virtually no rifampin killed 2.310.10 log_10_ CFU/mL below stasis the first 10 days of therapy, then failed as evidenced by the bacterial regrowth. The inadequate isoniazid exposure (C_max_ = 0.31 mg/L) combined with rifampin C_max_ of 3.99 mg/L (equivalent to 20 mg/kg dose accounting for the free drug concentration) killed 2.59 ± 0.57 log_10_ CFU/mL *Mtb* below stasis and therapy failed after study day 7.

Figure 1 shows the time-kill curves for the non-treated controls and two treatment regimens. We deliberately used a high inoculum of *Mtb* H37Rv (7.88 log_10_ CFU/mL) in anticipation of a preexisting drug-resistant subpopulation accounting for the mutation frequency of the study drugs (20). Maximum kill below stasis (day 0 bacterial burden) by Regimen 2 was 2.25 ± 0.28 log_10_ CFU/mL observed on day 10, with a linear kill rate of −0.23 ± 0.01 log_10_ CFU/mL/d. For Regimen 3, the maximum kill below stasis of 2.25 ± 0.28 log_10_ CFU/mL was observed on day 7 with a linear kill rate of −0.51 ± 0.08 log_10_ CFU/mL/d. The two slopes differed significantly from each other (*P* = 0.019). However, both regimens eventually failed to control the *Mtb* growth, and the bacterial burden was not statistically different compared with the non-treated controls on day 28 of the study.

Although no rifampin or isoniazid resistance in *Mtb* was observed using the drug-supplemented agar, SMOR detected heteroresistance, as shown in Table 1. The table lists the targeted mutations in *katG*, *rpoB*, and *gyrA* genes of *Mtb*, used in the SMOR analysis with the proportion of isoniazid, rifampin, and fluoroquinolone-resistant *Mtb* (based on the sequencing read counts) on study day 28. Notably, SMOR detected the presence of isoniazid and rifampin resistance in the inoculum; the proportion remained unchanged in the non-treated control HFS-TB units over 28 days of the study indicating no amplification of resistance or acquired AMR in the absence of drug pressure. In HFS-TB treated with Regimen 2 and Regimen 3, there was an amplification of drug-resistant populations. Mutations were detected in *katG* (S315N), *rpoB* (Q513K, H526N, and S531L), and *gyrA* gene (G88C, D94Y, and D94G) of *Mtb*, indicative of resistance to isoniazid, rifampin, and fluoroquinolones, respectively. It is worth mentioning that none of the HFS-TB units were treated with fluoroquinolones. Notably, the *rpoB* mutations arising from Regimen 2 (undetectable rifampin), which were at *rpoB Q513K* (new, not in inoculum or non-treated) and *rpoB H526N* (amplified but present in inoculum) differed from those that arose in Regimen 3 (adequate rifampin but low isoniazid AUCs), which were at *rpoB* S531L (amplified but present in inoculum).

**TABLE 1:** The proportion of drug-resistant *M. tuberculosis* emerged in the HFS-TB after suboptimal drug exposure over 28 days^*a*^

Regimen	Isoniazid	Rifampin			Fluoroquinolones		
	*katG* S315N	*rpoB* Q513K	*rpoB* H526N	*rpoB* S531L	*gyrA* D94Y	*gyrA* D94G	*gyrA* G88C
Inoculum	22/21070 (0.1%)		68/33700 (0.2%)	48/34036 (0.14%)			
Nontreated controls	21/20238 (0.1%) to 95/63875 (0.15%)		49/41246 (0.12%) to 62/22217 (0.28%)	36/22405 (0.16%)	73/32197 (0.23%)		107/83116(0.13%)
Regimen 2		74/42548 (0.17%)	418/42354 (0.99%) to 214/5774 (3.71%)				
Regimen 3				301/7620 (3.95%)		507/31086 (1.63%)	

^*a*^ There were three HFS-TB units per regimen. Data are presented as the number of reads mapped to a given nucleotide different from wild-type *M. tuberculosis* H37Rv divided by the total number of reads mapped to the position. Numbers in parentheses denote the percentage of reads representing the proportion of drug resistance. An empty cell means no sequencing reads to identify heteroresistance, i.e., wild-type sequence.

### HFS-TB study with fluoroquinolone-based combinations

For the drug-resistant clinical strain (SAMRC-11B) used in the HFS-TB experiments, the MICs were 8 mg/L for isoniazid, >0.25 mg/L for rifampin, >0.125 mg/L for moxifloxacin, and 2.0 mg/L for levofloxacin. This isolate was transformed into a semi-dormant bacteria (SDB) phenotype by acidifying the media to a pH of 5.8, as described previously (21). The HFS-TB units, with circulating acidified medium to maintain the SDB state of *Mtb*, were treated with isoniazid alone or isoniazid plus rifampin or rifampin plus moxifloxacin or rifampin plus levofloxacin. The isoniazid AUC_0-24_ achieved in the HFS-TB units based on sampling of the central compartment was 48.27 ± 0.74 mg*h/L (more than three times AUCs associated with amplifying AMR), rifampin AUC_0-24_ was 8.32 ± 1.53 mg*h/L (2.56 times higher AUC associated with amplifying AMR), moxifloxacin AUC_0-24_ was 100.9 ± 3.26 mg*h/L, and levofloxacin AUC_0-24_ was 137.5 ± 0.56 mg*h/L, respectively.

**Fig 2:**
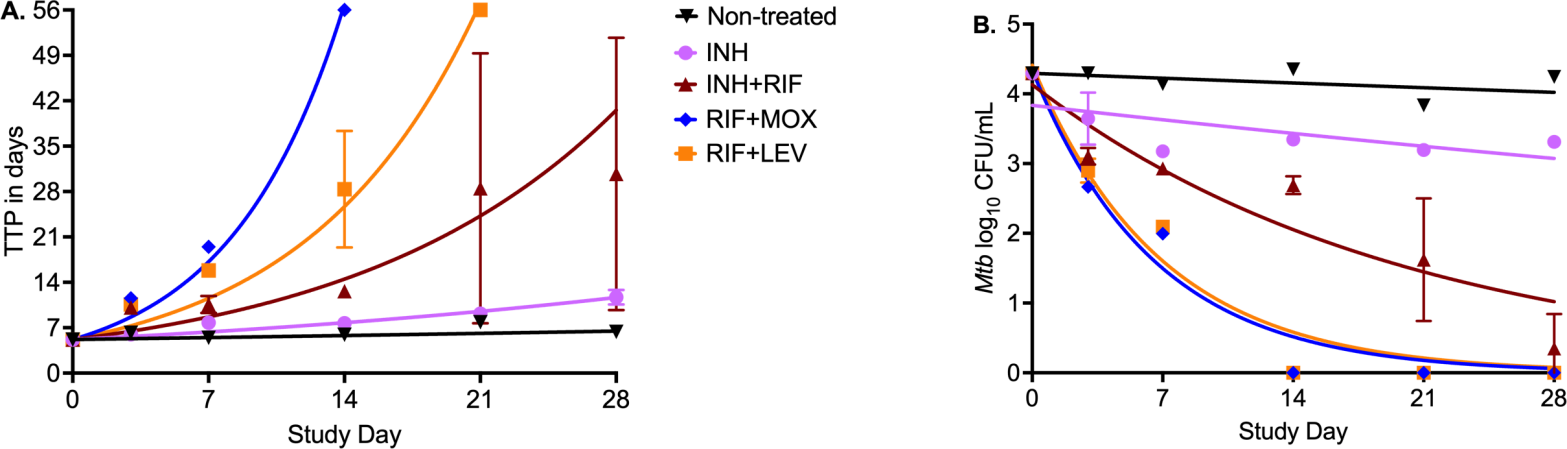
Evaluation of fluoroquinolones in combination with rifampin against MDR-TB *M. tuberculosis*. Symbols are mean and standard deviation and graphs are an exponential function. (**A**) TTP trajectories with different drug combinations showing isoniazid could still be effective, albeit at higher exposure. Based on exponential decline models, the elimination rate constants (unitless since its time/time) based in HFS-TB treated with isoniazid (INH) monotherapy were 0.029 (95% CI 0.026–0.032; r^2^ = 0.890), for isoniazid-rifampin (INH-RIF) 0.073 (95% CI: 0.065–0.081; r^2^ = 0.853), rifampin-moxifloxacin (RIF-MOX) 0.171 (95% CI: 0.164–0.178; r^2^ = 0.984), and rifampin-levofloxacin (RIF-LEV) 0.114 (95% Cl: 0.109–0.119; r^2^ = 0.953) but were 0.008 (95% Cl: 0.006–0.010; r^2^ = 0.872) for non-treated controls. The RIF-MOX regimen performed best of all regimens, followed by RIF-LEV, INH-RIF, and INH monotherapy performing worst in liquid cultures. RIF-MOX and RIF-LEV completely sterilized the HFS-TB units. (**B**) Using log_10_ CFU/mL, RIF-MOX performed the same as RIF-LEV; otherwise, the pattern was similar to the TTP readout. Based on the exponential decline models, the elimination rate constants (log_10_ CFU/mL/d) with INH monotherapy were 0.008 (95% CI: 0.001–0.015; r^2^ = 0.421), for INH-RIF 0.050 (95% CI: 0.032–0.072; r^2^ = 0.831), RIF-MOX 0.153 (95% CI: 0.120–0.193; r^2^ = 0.965), and RIF-LEV 0.145 (95% Cl: 0.113–0.185; r^2^ = 0.961) but was 0.002 (95% Cl: −0.001 to 0.006; r^2^ = 0.241) for non-treated.

Figure 2A shows the changes in the mycobacterial growth indicator tube (MGIT)-derived time-to-positive (TTP) with isoniazid alone or in combination with rifampin, moxifloxacin, and levofloxacin. The time-in-protocol was set to 56 days, and any MGIT tube that did not show a growth unit on day 56 was recorded as negative. The TTP of the inoculum was 2.28 days, higher than calculated day 0 which means bacterial burden dipped slightly after loading the SDB cultures into the peripheral compartment of the HFS-TB units. Based on the exponential decline model, the kill rate constants (*k_kill_*) shown in Fig. 2, the isoniazid-rifampin effect was more than double that of the isoniazid monotherapy, and rifampin-moxifloxacin achieved better *k_kill_* than the rifampin-levofloxacin combination. As a result of the faster kill, moxifloxacin-rifampin achieved sterilization 1 week ahead of the rifampin-levofloxacin combination. Figure 2B shows the time-kill curves based on the CFU/mL measurements at different time points over the 28-day study. Isoniazid monotherapy *k_kill_* was greater than zero, whereas that for non-treated controls was statistically zero. The rank order of *k_kill_* was similar to that with TTP readout, with the difference that moxifloxacin-rifampin was not statistically better than rifampin-levofloxacin.

Regarding AMR, the proportion of rifampin-resistant subpopulation in the inoculum was 1.24% on day 0. No CFU grew of rifampin-supplemented agar, including in the isoniazid monotherapy regimen. There were no moxifloxacin-resistant CFUs in the inoculum or any treatment regimens during the 28 days of the study. The proportion of levofloxacin-resistant CFU/mL in the inoculum was 0.05%, which increased to 3.66% by day 28 in the non-treated control systems. No levofloxacin resistance was observed in the systems treated with isoniazid-rifampin, rifampin-moxifloxacin, or rifampin-levofloxacin combinations.

## DISCUSSION

Tuberculosis treatment regimens are affected by the resistance status at the start of therapy. Therefore, it is important to detect the emergence of drug resistance as early as possible during therapy to alter the treatment regimen and help ensure a successful treatment outcome. Clinical outcomes and HFS-TB work have shown that intra-lesional concentrations and PK variability (and, therefore, dose) are the main drivers of both treatment failure and acquired AMR, likely more important than patient treatment compliance (7, 13–15, 22–24). Therefore, therapeutic drug monitoring should be considered to determine if the optimal drug exposure necessary for effective *Mtb* elimination is achieved with the given drug dose combination (23, 25–27).

Often, only the “critical concentration” of a drug is used to categorize the infecting strain as susceptible or resistant. However, to determine if the pharmacokinetics/pharmacodynamics (PK/PD) target is achieved with the given drug dose, MIC determination is needed. Many commercial molecular resistance tests can detect the presence of drug-resistant populations only when they reach 5%–65% of the total bacterial population (28, 29). However, by then, it is too late, given the limited number of effective anti-TB drugs. A recent analysis of retrospectively collected serial isolates from patients who transitioned from MDR-TB to pre-XDR-TB and/or XDR-TB during treatment identified the frequent presence of heteroresistance in patient samples 1–18 months before the detection of phenotypic resistance (11).

In the present study, we first showed that the high-resolution targeted sequencing SMOR method can successfully detect early and growing heteroresistance in *Mtb*. Second, we demonstrated that with continuous suboptimal isoniazid and rifampin exposures, *Mtb* developed genetic mutations not only to the drugs in the combination regimen but also to other effective second-line drugs bacteria were not exposed, such as fluoroquinolones. Our findings are in accordance with those of previous reports of cross-resistance to different classes of drugs as well as the “antibiotic resistance arrow of time” and the clinical evidence of the evolution of heteroresistance to resistant subpopulations replacing the susceptible population over the months of anti-TB therapy (11, 24, 30). The role of different rifampin exposures in the generation of different mutations indicates that the drug concentrations are the taut bowstring that launches the antibiotic resistance arrow of time and the determinant for where the arrow lands. The SMOR method provides a unique opportunity to intervene and alter the treatment regimen during the arrow’s flight, either by increasing drug dosage to achieve the optimal exposure (23) (provided a higher dosage will not be in the range known to cause adverse events) or by modifying the regimen to include drugs to which the infecting strain(s), including heteroresistant subpopulations, are still susceptible.

Finally, we showed that moxifloxacin and levofloxacin, when combined with rifampin, sterilized HFS-TB units infected with isoniazid mono-resistant clinical strain of *Mtb*. This finding reinforces the evidence regarding fluoroquinolone efficacy against different *Mtb* metabolic populations (slowly replicating bacilli at acidic pH in this study) and for moxifloxacin dosing in pan-TB treatment shortening regimens (31, 32). The regimens containing rifampin and fluoroquinolone were successful at suppressing drug-resistant *Mtb* growth, similar to a previous HFS-TB study (33) that used log-phase growth *Mtb* reporting synergy between moxifloxacin and rifampin with regard to the resistance suppression.

The potential limitation of our preclinical sterilizing effect HFS-TB study is that we relied only on CFU measurements to determine the evolution of drug resistance to rifampin, moxifloxacin, and levofloxacin. Longitudinal SMOR analysis could have informed on the developmental kinetics of resistance-associated genetic mutations, other than to isoniazid, capturing the heteroresistance dynamics before sterilization of the HFS-TB units.

To conclude, with suboptimal drug concentrations, therapy failure is near certainty due to the emergence of acquired drug resistance. Fluoroquinolones at optimal doses identified using the PK/PD studies can be used to treat mono-resistant and MDR-TB. Molecular techniques such as SMOR provide an opportunity to detect heteroresistance early to individualize the therapy and protect fluoroquinolones when added to the MDR-TB treatment regimen.

## MATERIALS AND METHODS

### Bacteria, materials, and reagents

Drug-susceptible laboratory strain *Mtb* H37Rv (ATCC #27294) and a drug-resistant clinical strain (SAMRC-11B) were used in the experiments. Isoniazid, rifampin, and pyrazinamide were purchased from BOC Sciences (New York, USA). Levofloxacin and moxifloxacin were purchased from the University of Texas Health Science Center at Tyler campus pharmacy. Cellulosic hollow fiber cartridges (C8008) were purchased from FiberCell (USA), and the MGIT liquid culture system and other supplies were purchased from Becton Dickinson, USA.

### Suboptimal drug exposure and heteroresistance in the HFS-TB

MIC of the study drugs was performed using the broth microdilution and MGIT method (12, 34). The detailed description of the HFS-TB has been published elsewhere (35, 36). Briefly, HFS-TB units were inoculated with 20 mL of ~1.5*10^6^ CFU/mL logarithmic phase growth cultures of drug-susceptible *Mtb* H37Rv. The HFS-TB units were treated with isoniazid, rifampin, and pyrazinamide for 28 days, with peak serum concentration (C_max_) we had observed in patients in a clinical trial of patients on treatment with the combination of isoniazid (300 mg/d), rifampin (600 mg/d), and pyrazinamide (1500 mg/d) who had failed the therapy due to the emergence of acquired AMR (7). Regimen 1 was non-treated controls, Regimen 2 was a combination of isoniazid C_max_ 7.7 mg/L plus rifampin C_max_ 0.02 mg/L plus pyrazinamide C_max_ 80 mg/L (i.e., inadequate rifampin), and Regimen 3 was a combination of (inadequate) isoniazid C_max_ 0.4 mg/L plus rifampin C_max_ 4.6 mg/L plus pyrazinamide C_max_ 80 mg/L. There were three replicate HFS-TB units per regimen. For a clinical context, the acceptable range of peak concentrations with a standard daily dose of 300 mg isoniazid ranges from 3 to 6 mg/L, for rifampin 600 mg ranges from 8 to 24 mg/L (free drug concentration ~20% of the total), and pyrazinamide 1500 mg ranges from 20 to 60 mg/L (23, 26, 37).

The circulating media in the HFS-TB was Middlebrook 7H9 broth supplemented with 2% dextrose at pH 6.8 (13, 38). The total volume of the HFS-TB, including the flow path of the cartridge, was 152 mL, and fresh media inflow and waste media outflow rates, using silicon-coated 14 gauge tubing, were set to 0.6 mL/min. Drugs were infused using programable syringe pumps, using 13 gauge silicon coated tubing, into the central compartment of the HFS-TB units, excluding the non-treated controls. Systems were sampled before drug infusion (0 h) followed by 3, 6, 9, 15, and 23.5 h to measure the drug concentrations using LC-MS/MS (13). The measured concentrations were used to calculate the drug exposure achieved in the HFS-TB units for each drug in the combination. The peripheral compartments were sampled on days 0, 7, 14, 19, and 28 to enumerate the bacterial burden. Samples were washed twice with normal saline to remove the carryover drug, 10-fold serially diluted, and inoculated on Middlebrook 7H10 agar supplemented with 10% oleic acid-dextrose-catalase (OADC). The cultures were incubated at 37°C under 5% CO_2_, and colonies were counted after 21 days of incubation. To determine the drug-resistant subpopulation sizes, agar was supplemented with 3× MIC and incubated for up to 6 weeks before colonies were counted.

### Detection of heteroresistance

SMOR analysis was performed to detect the heteroresistance and compare the proportion of drug-resistant *Mtb* estimated by the conventional agar-based method. Briefly, DNA was extracted from the day 28 HFS-TB samples and subjected to sequencing library preparation and subsequent analysis of the sequencing reads (10, 11, 39). Table S1 lists the target genes and mutations conferring resistance to isoniazid and rifampin, and additionally, *gyrA and* gyr*B* mutations associated with the non-study drugs such as fluoroquinolones. The reads were aligned to the reference *Mtb* genome (NC_000972), and variant calls were made for nucleotide changes in the drug-resistance genes included in the SMOR analysis using the Amplicon Sequencing Analysis Pipeline tool (9).

### Efficacy of fluoroquinolones in the presence of preexisting isoniazid resistance

In the sterilizing effect HFS-TB study, to evaluate the efficacy of moxifloxacin and levofloxacin in the presence of preexisting isoniazid resistance in *Mtb*, we used an isoniazid-resistant clinical strain (SAMRC-11B) with mutations as listed in Table S2. Transformation of the log-phase growth cultures to semi-dormant bacilli (SDB) at an acidic pH of 5.8 was performed as described previously (21). Duplicate HFS-TB units were treated with a human equivalent dose of (i) isoniazid 450 mg/d alone, (ii) isoniazid in combination with rifampin 600 mg/d, (iii) rifampin plus moxifloxacin (800 mg/d), or (iv) rifampin plus levofloxacin (1,500 mg/d). Isoniazid monotherapy was tested to determine if resistance can be overcome with a higher drug exposure, provided it does not cause toxicity. The moxifloxacin and levofloxacin doses were selected based on the recently published WHO Technical report on PK/PD of anti-TB drugs (40). Nontreated HFS-TB units served as the growth control. Sampling of the peripheral compartments for the drug concentration measurements and the central compartments for the estimation of total and drug-resistant *Mtb* subpopulation was performed as described above. The cultures were incubated at 37°C under 5% CO_2_, and colonies were counted after 21 days of incubation. To determine the drug-resistant subpopulation sizes, agar was supplemented with 3× MIC and incubated for up to 6 weeks before the colonies were counted. A portion of the processed sample was inoculated into the MGIT tubes to record the TTP using EpiCenter software (Becton Dickinson, USA).

### LC-MS/MS and PK/PD data analysis

The drug concentrations in the HFS-TB samples were measured using the previously published methods (13, 41, 42) and modeled using WinNonLin (Phoenix, Certara, NJ, USA). Linear regression and exponential decline model were used to calculate the kill rate with each treatment regimen. GraphPad Prism was used for statistical analysis and data graphing.

## Data Availability

The raw data for the results presented in the article are available from the corresponding author upon reasonable request.

## SUPPLEMENTAL MATERIAL

Supplemental tables aac.01084-24-s0001.docx Tables S1 and S2.

ASM does not own the copyrights to Supplemental Material that may be linked to, or accessed through, an article. The authors have granted ASM a non-exclusive, world-wide license to publish the Supplemental Material files. Please contact the corresponding author directly for reuse.
