# Supplementary material for: Fluoroquinolones and rifampin combination in the backdrop of heteroresistant tuberculosis
Source: Antimicrob Agents Chemother. 2025 Jan 16;69(2):e01084-24. doi: 10.1128/aac.01084-24 (PMC11823603; doi:10.1128/aac.01084-24)
Supplement: Supplemental tables — Tables S1 and S2. [file aac.01084-24-s0001.docx]

**Fluoroquinolones and rifampin combination in the backdrop of heteroresistant tuberculosis**

Vanessa B. Vogensen^1#^, Sanjay Singh^2#^, Christopher J Allende^4^, David M Engelthaler^4^, Gunavanthi D. Boorgula^2^, Tania A. Thomas^5^, Marieke GG Sturkenboom^6^, Onno W. Akkerman^1,7^, Tawanda Gumbo^3§^, Shashikant Srivastava^2,8*^

#Joint first authors

^1^Department of Pulmonary Diseases and Tuberculosis, University Medical Center Groningen, The Netherlands.

^2^Division of Infectious Diseases, Department of Medicine, University of Texas at Tyler School of Medicine, Tyler, Texas, USA.

^3^Quantitative Preclinical & Clinical Sciences Department, Praedicare Inc., Dallas, Texas.

^4^Translational Genomics Research Institute, Flagstaff, Arizona, USA.

^5^Department of Medicine, University of Virginia, Charlottesville, Virginia.

^6^Department of Clinical Pharmacy and Pharmacology, University Medical Centrum Groningen, University of Groningen, Groningen, The Netherlands.

^7^TB Center Beatrixoord, Haren, University Medical Center Groningen, Groningen, The Netherlands.

^8^Department of Cellular and Molecular Biology, University of Texas Health Science Centre at Tyler, Tyler, Texas, USA.

***Corresponding author:**

Shashikant Srivastava, Ph.D.

Department of Medicine, UT Tyler School of Medicine,

11937 US Highway 271, Tyler, TX, 75708, USA

e-mail: [shashi.kant@uthct.edu](mailto:Shashi.kant@uttyler.edu); Phone: +1-903-877-7684

^§^**Co-corresponding author:**

Tawanda Gumbo, MD

Praedicare Inc.,

14830 Venture Drive,

Dallas, Texas 75234

e-mail: [rozvi1@praedicareinc.com](mailto:rozvi1@praedicareinc.com)

**Supplementary Table 1. List of target amino acid positions to detect isoniazid, rifampin, and fluoroquinolone heteroresistance in HFS-TB using SMOR analysis.**

| **Isoniazid (*katG*)** | **Rifampin (*rpoB*)** | **Fluoroquinolones (*gyrA*)** |
| --- | --- | --- |
| S315N | L511P | A90V |
| S315T | Q513K | D89N |
|  | Q513L | D94A |
|  | Q513P | D94G |
|  | D516A | D94H |
|  | D516F | D94N |
|  | D516G | D94Y |
|  | D516V | G88A |
|  | D516Y | G88C |
|  | H526C | S91P |
|  | H526D |  |
|  | H526G |  |
|  | H526L |  |
|  | H526N |  |
|  | H526R |  |
|  | H526Y |  |
|  | S522L |  |
|  | S522Q |  |
|  | S531F |  |
|  | S531L |  |
|  | S531W |  |
|  | L533P |  |

**Supplementary Table 2. Mutation profile of the isoniazid-resistant *M. tuberculosis* clinical strain used in the HFS-TB study.**

| **Gene** | **Position** | **Reference** | **Allele** | **Amino acid change** | **Product** |
| --- | --- | --- | --- | --- | --- |
| *kat*G | 2154724 | C | A | Arg463Leu | catalase-peroxidase-peroxynitritase |
| *kat*G | 2155168 | C | G | Ser315Thr | catalase-peroxidase-peroxynitritase |
| *emb*C | 4240172 | G | A | Val104Met | indolylacetylinositol arabinosyltransferase |
| *emb*C | 4241562 | G | A | Arg567His | indolylacetylinositol arabinosyltransferase |
| *emb*C | 4242075 | G | A | Arg738Gln | indolylacetylinositol arabinosyltransferase |
| *emb*R | 1416210 | T | C | Ile380Val | transcriptional regulatory protein EMBR |
| *emb*R | 1416213 | G | C | Gln379Glu | transcriptional regulatory protein EMBR |
| *emb*R | 1416222 | A | G | Phe376Leu | transcriptional regulatory protein EMBR |
| *emb*R | 1416234 | A | C | Cys372Gly | transcriptional regulatory protein EMBR |
| *gyr*A | 7362 | G | C | Glu21Gln | DNA gyrase subunit A |
| *gyr*A | 7585 | G | C | Ser95Thr | DNA gyrase subunit A |
| *gyr*A | 9304 | G | A | Gly668Asp | DNA gyrase subunit A |
| *gid*B | 4407904 | G | A | Ser100Phe | 16S rRNA methyltransferase GidB |

**LC-MS/MS analysis**

The drug concentrations in the HFS-TB samples were measured using the previously published methods (13, 23, 50) and modeled using WinNonLin (Phoenix, Certara, NJ, USA). Briefly, standards were purchased from BOC Sciences, (NY, USA) and internal standards (IS) were purchased from CDN Isotopes (Quebec, Canada). LC-MS/MS analysis was performed using Waters Acquity UPLC coupled with Waters Xevo TQ mass spectrometer (Milford, MA). Data was collected using MassLynx version 4.1 SCN810 software. Separation was achieved on a Waters Acquity UPLC HSS T3 column (50 x 2.1 mm; 1.8 μm). All standard and internal standard (IS) stock solutions were prepared at 1 mg/mL in 80:20 methanol:water and stored at -20ºC. Calibration curve, and low- and high-quality control samples (LQC and HQC) were prepared by diluting the stock solution in the blank medium. In a 96-well plate, samples were diluted 1:20 with IS solution in 0.1% aqueous formic acid (FA). The plates were vortexed, and loaded on the instrument, and two microliters of the samples were injected. The mobile phase was a gradient mixture of (A) 0.1% aqueous formic acid, and (B) 0.1% formic acid in methanol. Compounds were detected using ESI in MRM mode. The inter-and intraday percentage coefficient of variation (%CV) were ranged from 3% to 13%.The measured drug concentrations were used to calculate peak concentration (C_max_) and 24 h area under the concentration-time curve (AUC_0-24_) with each drug. GraphPad Prism was used for statistical analysis and graphing of the data.
